# Cardiac Safety of TGF-β Receptor I Kinase Inhibitor LY2157299 Monohydrate in Cancer Patients in a First-in-Human Dose Study

**DOI:** 10.1007/s12012-014-9297-4

**Published:** 2014-12-09

**Authors:** Richard J. Kovacs, Giuliana Maldonado, Analia Azaro, Maria S. Fernández, Federico L. Romero, Juan M. Sepulveda-Sánchez, Mary Corretti, Michael Carducci, Melda Dolan, Ivelina Gueorguieva, Ann L. Cleverly, N. Sokalingum Pillay, Jose Baselga, Michael M. Lahn

**Affiliations:** 1 Krannert Institute of Cardiology, Indiana University School of Medicine, Indianapolis, IN 46202 USA; 2 Department of Cardiology, Vall d′Hebron, Barcelona, Spain; 3 Medical Oncology, Vall d′Hebron, Barcelona, Spain; 4 Department of Cardiology, Hospital Universitario 12 de Octubre, Madrid, Spain; 5 Oncology, Hospital Universitario 12 de Octubre, Madrid, Spain; 6 Division of Cardiology, Johns Hopkins University, Baltimore, MD 21218 USA; 7 Johns Hopkins Kimmel Cancer Center, Baltimore, MD 21218 USA; 8 Department of Cardiology and Cardiovascular Disease, Saint Louis University Hospital, St. Louis, MO 63103 USA; 9 Eli Lilly and Company, Erl Wood, UK; 10 Eli Lilly and Company, Building 31/4, 893 S. Delaware St., Indianapolis, IN 46285 USA

**Keywords:** LY2157299, Cardiac safety, Glioma, First-in-human dose study

## Abstract

Transforming growth factor-beta (TGF-β) signaling plays an important role in the fetal development of cardiovascular organs and in the repair mechanisms of the heart. Hence, inhibitors of the TGF-β signaling pathway require a careful identification of a safe therapeutic window and a comprehensive monitoring of the cardiovascular system. Seventy-nine cancer patients (67 glioma and 12 solid tumor) enrolled in a first-in-human dose study and received the TGF-β inhibitor LY2157299 monohydrate (LY2157299) as monotherapy (*n* = 53) or in combination with lomustine (*n* = 26). All patients were monitored using 2D echocardiography/color and Spectral Doppler (2D Echo with Doppler) every 2 months, monthly electrocardiograms, thorax computer tomography scans every 6 months, and monthly serum brain natriuretic peptide (BNP), troponin I, cystatin C, high-sensitivity C-reactive protein (hs-CRP). Administration of LY2157299 was not associated with medically relevant cardiovascular toxicities, including patients treated ≥6 months (*n* = 13). There were no increases of troponin I, BNP, or hs-CRP or reduction in cystatin C levels, which may have been considered as signs of cardiovascular injury. Blood pressure was generally stable during treatment. Imaging with echocardiography/Doppler showed an increase in mitral and tricuspid valve regurgitation by two grades of severity in only one patient with no concurrent clinical symptoms of cardiovascular injury. Overall, this comprehensive cardiovascular monitoring for the TGF-β inhibitor LY2157299 did not detect medically relevant cardiac toxicity and hence supports the evaluation of LY2157299 in future clinical trials.

## Introduction

Transforming growth factor-beta (TGF-β) signaling plays an important role in the development of cardiovascular organs and is also a key regulator of cardiovascular remodeling after injury [1, 2]. Its important role in ontogenesis of the heart was identified by knocking out TGF-β signaling proteins [3]. TGF-β ligands (TGF-β1, TGF-β2, and TGF-β3) regulate diverse biological functions [4]. All three ligands can bind to a specific receptor by first engaging the TGF-β receptor type I (TGF-βRI or ALK5), which then heterodimerizes with the TGF-βRII. This heterodimer complex phosphorylates the intracellular proteins SMAD2 and SMAD3, which initiate an activation cascade to induce several nuclear transduction proteins. By knocking out either the ligands or the SMAD proteins, the development of the heart can be blunted and can lead to intrauterine death [5].

In cardiovascular disease, TGF-β signaling has been associated with remodeling of the heart after myocardial infarction and its overexpression has been associated with heart failure [6]. Preclinical models have shown that blocking TGF-β signaling with pharmacological agents can prevent injury-induced cardiomyopathies and their associated pathologies [7]. In vessels, TGF-β signaling regulates inflammatory responses of the endothelium and smooth muscle to injury [8]. Shear/stress stimuli in the vessels are one of the key inducers of TGF-β signaling [9]. This activation of TGF-β signaling can prevent aneurysm formation, but is present in aneurysms despite a loss of function in the TGF-β signaling pathway [10]. Such a condition is observed in patients with Loeys–Dietz Syndrome (LDS), a subset of Marfan Syndrome, where patients present with aortic dilatation and thoracic aneurysms [11]. In such patients and also in animal models mimicking the condition of LDS patients, TGF-β signaling is induced, despite the genetic evidence in loss of function. Toxicology studies with TGF-β inhibitors that block TGF-β RI/ALK5 have reported toxicities that share the cardiovascular findings in LDS patients [12]. Such findings included structural changes of the heart valve and aneurysms of the ascending aorta and aortic arch. These observations have discouraged the clinical development of small molecule inhibitors. LY2157299 monohydrate (LY2157299) is a TGF-βRI kinase inhibitor that interrupts the receptor-mediated signaling cascade. Hence, it was critical to develop a therapeutic window for the safe administration of LY2157299 during a first-in-human dose (FHD) study [13, 14]. During the FHD study, the administration of LY2157299 was accompanied with a comprehensive and prospective cardiac safety monitoring. Cardiac safety evaluations including echocardiography/Doppler, plasma markers of cardiac function, electrophysiological evaluations, and radiographic imaging to assess the ascending aorta and aortic arch for aneurysm formation were used.

The results of this FHD study of LY2157299 are described herein. Overall, the selected dose range of LY2157299 was safe, and no significant cardiac toxicities were observed. This monitoring approach is currently being extended to all other ongoing trials with LY2157299.

## Methods

### Patients

As previously described [13], patients who had a histologic or cytologic diagnosis of cancer for which no proven effective therapy existed were included in the first two cohorts. Starting with cohort 3, only patients with relapsed and progressive glioblastoma were eligible for this study. All patients had to have disease that was measurable or non-measurable as defined by the Response Evaluation Criteria in Solid Tumors (RECIST) and for patients starting on cohort 3 onward as defined by Macdonald criteria [15]. All patients had to have performance status of ≤2 on the Eastern Cooperative Oncology Group (ECOG) scale. Patients were required to have adequate hematologic, hepatic, and renal function, and discontinued all previous therapies for cancer at least 4 weeks prior to the study enrollment. Exclusion criteria included medically uncontrolled cardiovascular illness and medically significant electrocardiogram (ECG) anomalies. Patients who had major abnormalities documented by echocardiography/Doppler, such as moderate or severe heart valve function defect and/or left ventricular ejection fraction (LVEF) of ≤50 %, were excluded from the study. Patients with tricuspid (trace or mild), pulmonary, mitral (trace or mild), or aortic (trace or mild) regurgitation by Doppler techniques were allowed to enter the study.

### Study Design

The study was conducted according to the principles of good clinical practice, applicable laws and regulations, and the Declaration of Helsinki. Each institution’s review board approved the study and all patients signed an informed consent document before study participation.

**Fig. 1:**
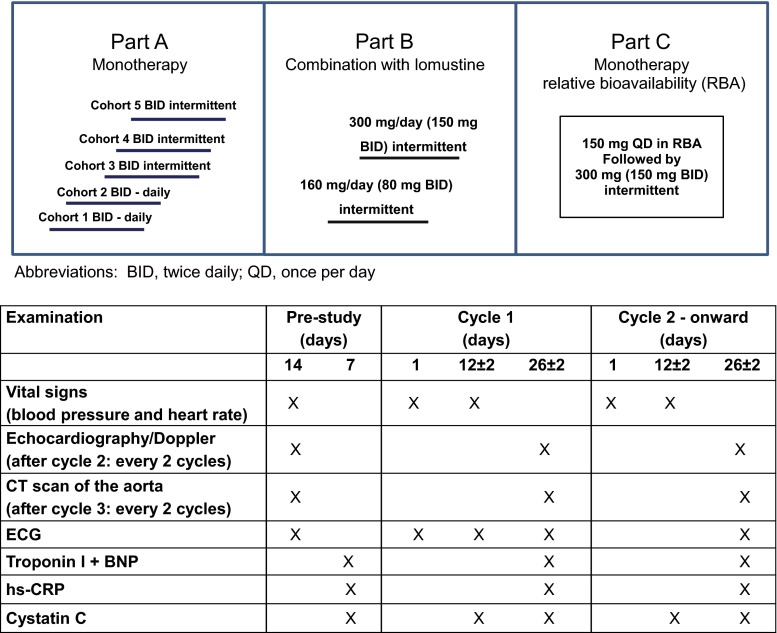
Study design of the first-in-human dose study with LY2157299 and timing of the cardiovascular assessments

LY2157299 was evaluated in a multicenter, open-label, non-randomized, dose escalation First-in-Human Phase 1 study (Fig. 1). There were three parts in the study: Part A was a dose escalation and enrolled patients with advanced or metastatic cancer and then only glioblastoma patients for the remainder of the study. Doses were escalated to a predetermined top dose of 300 mg/day [13]. Each cohort enrolled at least 3 patients and the number of patients per cohort was adjusted based on the pharmacokinetic (PK) profile and variability. In Part B, LY2157299 was combined with lomustine at two doses, 160 and 300 mg/day. Part C was designed as a relative bioavailability (RBA) study; all patients remained on study treatment after the RBA phase was completed and were then dosed with 300 mg/day LY2157299 monotherapy.

### Treatment

LY2157299 was given orally at doses of 20, 40, 80, 120, and 150 mg twice daily as a tablet in the morning and evening. Patients in the first two cohorts received LY2157299 daily as part of a 28-day cycle. Starting with cohort 3, all patients received LY2157299 on Day 1–14 of each 28-day cycle. No dose adjustments or reductions were allowed.

### Safety Assessments

**Fig. 2:**
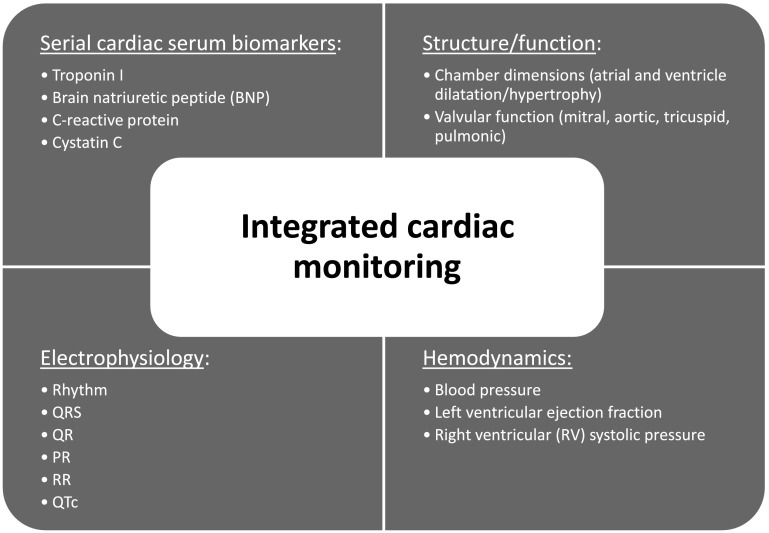
Overview of the parameters of the cardiovascular monitoring implemented during the first-in-human dose study with LY2157299

Safety was evaluated in patients who received at least one dose of LY2157299. Safety assessment was based on the summaries of adverse events (AEs) including severity (as defined by Common Terminology Criteria for Adverse Events version 3.0 (CTCAE, v3.0) and possible relationship to study drug, dose-limiting toxicities (DLTs) and laboratory changes at each dose level. Standard laboratory tests included chemistry, hematology, and urinalysis panels. Safety was also analyzed by ECG using a standardized assessment [16], echocardiography/Doppler [17], and additional laboratory tests specifically linked to cardiac toxicity, such as brain natriuretic peptide (BNP), troponin I, high-sensitivity C-reactive protein (hs-CRP), and cystatin C [18] (Figs. 1, 2). Besides the assessment of acute DLT detailed below, serum monitoring for cardiotoxicity was carried out beyond Cycle 1. Assessments were performed at the end of every other cycle starting at Cycle 2, except for cystatin C and hs-CRP which were taken at the end of every cycle, until study treatment discontinuation. Computer tomography (CT) scans or magnetic resonance images (MRI) of the thoracic aorta specifically assessing potential aneurysms of the ascending aorta and aortic arch were performed based on institutional imaging procedures [19]. Any clinically significant abnormal results were recorded as AEs. If there were clinically significant cardiac findings at discontinuation, echocardiography/Doppler and ECGs were to be repeated every 2 months for 6 months. In the event of no cardiac findings at study treatment discontinuation, one more echocardiography and ECG were planned after 2 months, unless the patient had started another treatment.

### 2D Echocardiography/Color Spectral Doppler

**Fig. 3:**
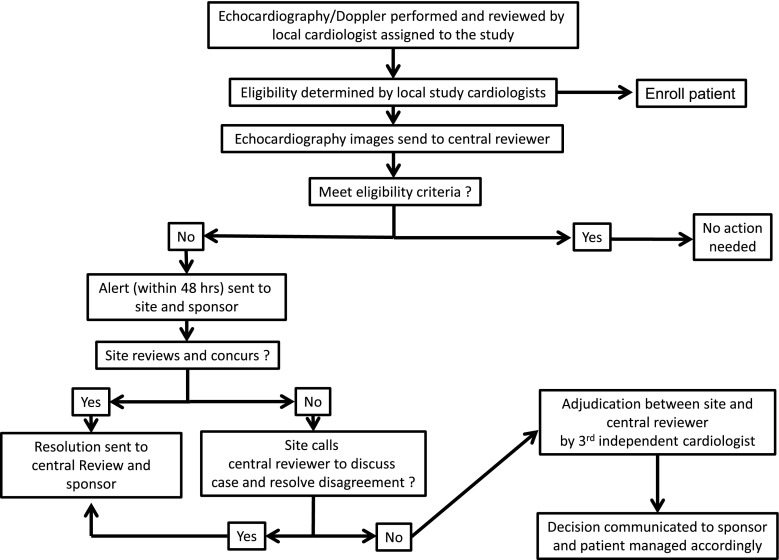
Alert and adjudication process of echocardiography/Doppler assessments between local and central echocardiography readers

Echocardiography/Doppler were performed according to a standard protocol and interpreted as per recommendations of echocardiography societies [17]. All images were submitted to a central echocardiologist who reviewed all images based on pre-specified variables (Table 1). During the study, an echocardiography alert process was implemented in order to ensure close monitoring of every patient, and reconciliation was pre-specified between local and central echocardiography review to ensure consistent grading of abnormality (Fig. 3).

**Table 1:** List of cardiac markers assessed

	Normal ranges
Serum biomarkers	
Troponin I	0–0.3 µg/L
BNP	0 ≤ age < 45: 2.7–33 ng/L 45 ≤ age ≤ 54: 2.7–46.7 ng/L 55 ≤ age < 65: 2.7–53.2 ng/L 65 ≤ age < 75: 2.7–72.3 ng/L 75 ≤ age < 111: 2.7–176 ng/L
hs-CRP	0–3 mg/L
Cystatin C	0.53–0.95 mg/L
Echocardiography/Doppler	
Continuous variables (normal range)	
LV internal dimension (diastolic)	≤2.8 cm/m^2^
LA volume (end-systolic)	≤36 mL/m^2^
LA dimension (end-systolic)	1.9–4.0 cm
LV ejection fraction	≥50 %
LV mass	≤115 g/m^2^—male ≤99 g/m^2^—female
PA systolic pressure	≤40 mmHg
Pulmonary flow velocity acceleration time	≥120 ms
Mitral deceleration time	≥160 ms and ≤220 ms
Mitral E/A ratio	≥0.75 and ≤1.5
E/Em	<10
Systolic blood pressure	Measurements in mmHg
Diastolic blood pressure	Measurements in mmHg
Semi-quantitative variables (severity scale)	
RA dilation	Normal, mild, moderate, severe
RV dilation	Normal, mild, moderate, severe
Mitral regurgitation	Absent, trace, mild, moderate, severe
Aortic regurgitation	Absent, trace, mild, moderate, severe
Pulmonic regurgitation	Absent, present
Tricuspid regurgitation	Absent, present
Wall motion	Normal/abnormal
Pericardial effusion	Absent, small, moderate, large
Electrocardiogram	
Standard measurements	
Hemodynamic measurements	
Blood pressure	90/60–140/90 mmHg
Heart rate	50–100 beats/min

Per-protocol attachment E/Em < 15 not <10 as in worksheet. Mitral and aortic valve area for evidence of stenosis should not be less than 2 and 1.5 cm/m^2^, respectively, but data were not collected

*BNP* brain natriuretic peptide, *hs*-*CRP* high-sensitivity C-reactive protein, *LA* left aortic, *LV* left ventricle, *PA* pulmonary artery, *RA* right aortic, *RV* right ventricle

#### DLT Assessment

Dose escalation to the second cohort proceeded after three patients completed one treatment cycle without a DLT and after careful assessment of their PK and safety information. Dose escalation to each subsequent dose was based on a combination of the number of DLTs at the dose tested, aggregate safety profile, and predicted exposure of area under the curve. Hematologic or non-hematologic toxicity with grade ≥3 was considered as a DLT in patients treated with study medication at different dose levels according to the National Cancer Institute and the CTCAE, v3.0. Specific DLT criteria for cardiac parameters were defined to be an increase of one or more grades of the semi-quantitative valvular insufficiency, left ventricular function, or right ventricular systolic pressure (normal, mild, moderate, or severe based on local laboratory limits). If a patient was normal at baseline and after the first cycle increased to mild, then a repeat echocardiography should be performed after 14 days. If results confirmed mild grade at repeat echocardiography, then patient was to be discontinued. If findings were normal, then the patient continued treatment per study protocol. In addition, an increase in left atrial or ventricular chamber size of 2 and 1 cm, respectively, or any evidence of damage to the heart’s large vessels from the CT scan would also be considered a DLT. For the serum cardiac safety markers, concentrations of BNP ≥ 3 times the baseline value and above the upper limit of normal (ULN) and sustained at two consecutive scheduled blood draws, and/or concentrations of troponin I above the ULN were also considered DLTs.

#### Statistical Analysis

The primary objective of this FHD study was to determine a safe and tolerable dose for future Phase 2 studies. This evaluation included assessment of cardiovascular toxicity (see DLT definition above). Consistent with the traditional DLT assessment in FHD trials of cancer patients, the study used descriptive statistical analyses and was not powered to determine statistically important differences between a standard treatment and the novel treatment with LY2157299. All summary tables and figures are given by monotherapy and combination therapy. Demographics, concomitant medication, and AEs were summarized using frequencies or summary statistics as appropriate. Shift tables to summarize maximum changes in severity after dosing were created for overall ECG evaluation. The frequency of patients experiencing increases from baseline in QTcF were listed by category: 0–30, >30–60, and >60 ms. For valvular regurgitation parameters, all measurements for patients who experienced an increase in severity of at least one grade are listed, together with the time-matched systolic blood pressure. This detail was provided in order to help interpret the event. Line plots over time for serum measurements and ECG parameters (together with normal limits where appropriate) and box–whisker plots for blood pressure and left ventricular fraction are provided.

## Results

A total of 79 patients were enrolled into this FHD study from 2006 to 2012, which included a period of 2 years when the study was placed on clinical hold awaiting new animal toxicology data. The majority of the patients were male in Parts A and B of the study; in Part C, there were more females (Table 2). Most patients were younger than 60 years. In Part A, there was a higher proportion of patients who had either a lower grade glioma or secondary glioma compared to patients enrolled in Parts B and C. Overall, most patients had a good performance status (ECOG 0 or 1) at the time of their cancer progression and after failing to respond to previous effective anticancer therapies (Table 2). Because of the entry criteria, all patients had an unremarkable cardiac function prior to the study entry.

**Table 2:** Patient characteristics and concomitant cardiovascular medication

Characteristics	Part A *N* = 39	Part B *N* = 26	Part C *N* = 14
Age (years)			
Mean (SD)	51.8 (14.88)	44.5 (10.35)	59.8 (12.74)
Median (range)	54.0 (22, 77)	43.5 (25, 61)	56.5 (34, 76)
Sex [*n* (%)]			
Male	30 (76.9)	19 (73.1)	5 (35.7)
Female	9 (23.1)	7 (26.9)	9 (64.3)
Origin [*n* (%)]			
Caucasian	39 (100)	24 (92.3)	14 (100)
Hispanic	–	1 (3.8)	–
West Asian	–	1 (3.8)	–
ECOG [*n* (%)]			
0	15 (38.5)	3 (11.5)	4 (28.6)
1	19 (48.7)	17 (65.4)	8 (57.1)
2	5 (12.8)	6 (23.1)	2 (14.3)
Glioma [*n* (%)]	*n* = 30^a^	*n* = 26	*n* = 9
Low grade, Grade II–III	9 (30.0)	4 (15.4)	2 (22.2)
Secondary, Grade IV	5 (16.7)	2 (7.7)	–
Primary, Grade IV	16 (53.3)	20 (76.9)	7 (77.8)

Drug class generic name	Patients (*n*)		
Cardiovascular drugs			
Alpha and beta blocking agents			
Labetalol	1		
Atenolol	1		
Bisoprolol	2		
Metoprolol tartrate	1		
Angiotensin-converting enzyme inhibitors			
Enalapril	6		
Lisinopril	1		
Angiotensin II receptor blockers			
Losartan	2		
Valsartan	2		
Olmesartan	1		
Antiarrhythmics			
Lidocaine	1		
Flecainide acetate	1		

*ECOG* Eastern Cooperative Oncology Group, *SD* standard deviation

^a^Data for two patients are not available

### Concomitant Cardiac Medications

The low number of cardiac medications suggested that patients had relatively good cardiovascular statuses (Table 2). The most common cardiac medication was enalapril (6/79, 7.6 %) followed by angiotensin II receptor blockers (5/79, 6.3 %).

### Safety Measures: Treatment-Emergent Adverse Events

Of 79 patients dosed, 37 died during the study—36 due to tumor progression and one related to pneumonia. One patient (in Part A in the 300 mg/day cohort) was identified as having a DLT of grade 4 thrombocytopenia. The patient completed Cycle 2 but died due to disease progression before he fully recovered from this event. In the entire study, 53 patients (53/79; 67 %) experienced at least one grade 3 or 4 adverse event, of which the events of 13 (13/79; 16.4 %) patients were possibly related to study treatment (Table 3). Two patients (2/79; 2.5 %) discontinued from study treatment due to platelet reduction (CTCAE v3.0 grades 2 and 4), both considered as possibly related to study treatment.

**Table 3:** Summary of number of patients with TEAEs and CTCAE severity grade by study part and study treatment relatedness

Study part	TEAEs		Related to study treatments	
	≥1 TEAE *n* (%)	≥1 Grade 3/4 *n* (%)	≥1 TEAE *n* (%)	≥1 Grade 3/4 *n* (%)
Part A (*N* = 39)	37 (95)	24 (62)	10 (26)	3 (8)
Part B (*N* = 26)	26 (100)	22 (85)	15 (58)	8 (31)
Part C (*N* = 14)	14 (100)	7 (50)	3 (21)	2 (14)
Total (*N* = 79)	77 (97)	53 (67)	28 (35)	13 (16)

Study treatment relatedness in Part B is to either LY2157299, lomustine or both

*CTCAE* common terminology criteria for adverse events, *TEAEs* treatment-emergent adverse events

### Plasma/Serum Markers to Assess Cardiac Function

**Fig. 4:**
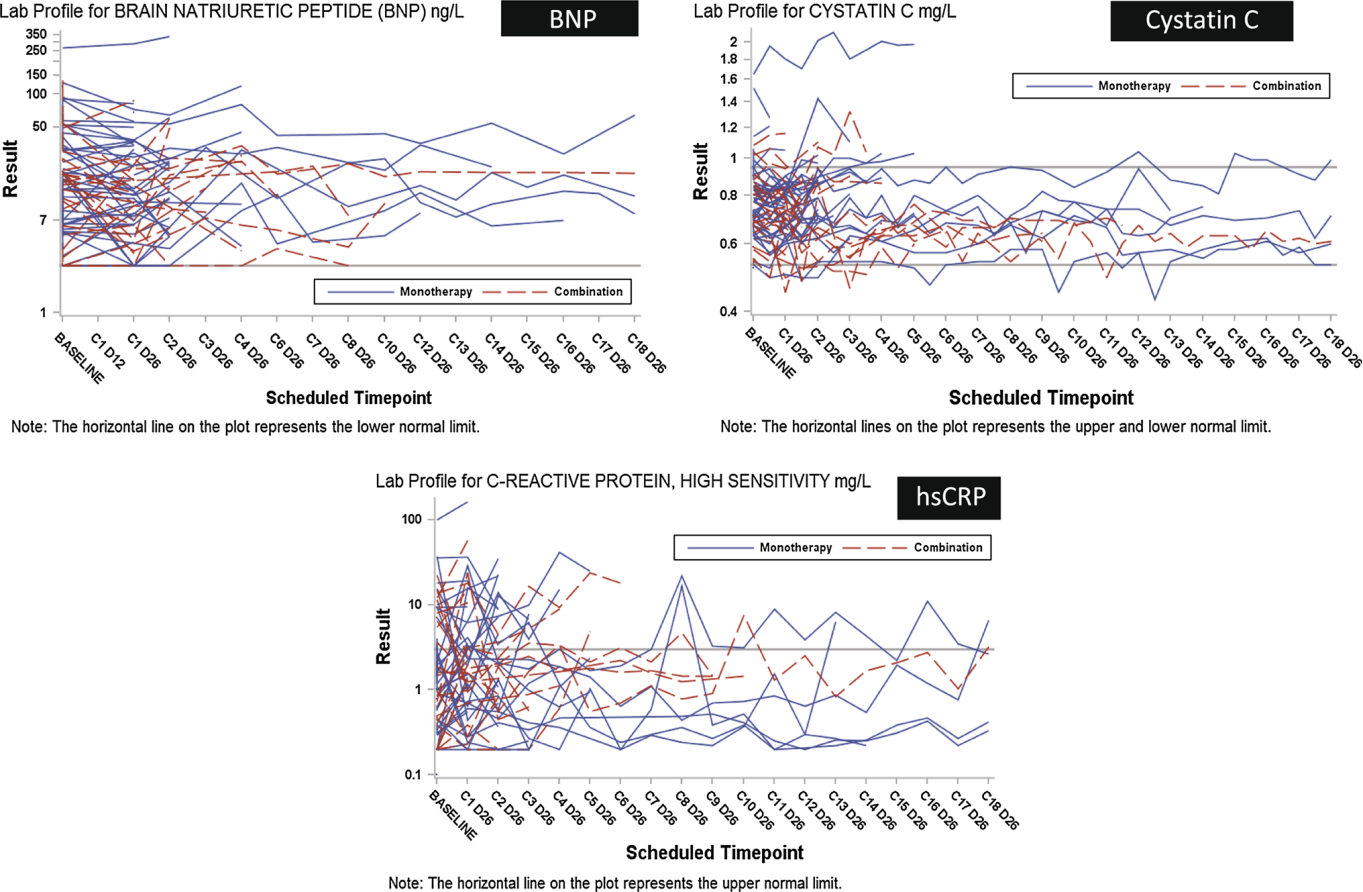
Serum cardiac markers and changes over time for brain natriuretic factor (BNP), cystatin C and high-sensitive C-reactive protein (hs-CRP). *Horizontal lines* reflect the norm values. *Blue lines* represent patients who received LY2157299 as monotherapy and *red* for patients who were treated with the combination of LY2157299 and lomustine

We used the markers troponin I, BNP and hs-CRP to serially evaluate for myocardial necrosis (troponin I), cardiac failure (BNP), or an inflammatory response (hs-CRP) (Fig. 4). One patient on monotherapy had higher than normal troponin I values on Days 122 and 127 post-dose. On both occasions, the recorded value was 0.06 ng/L (ULN = 0.05 ng/L). The patient discontinued because of progressive disease on Day 149 and values returned to normal on Day 156. No values were greater than the ULN in patients treated in combination with lomustine (data not shown). For BNP, no patients on monotherapy met the pre-specified toxicity criteria. One patient on combination therapy had concentrations that did not meet the pre-specified toxicity criteria and the BNP levels increased 16-fold from 3.8 ng/L at baseline to 59.0 ng/L by Day 56 (ULN age-adjusted was 53.2 ng/L). This patient discontinued from study treatment on Day 64 due to tumor progression (Fig. 4). There were some isolated instances of increased hs-CRP, but none were sustained over time. The isolated increases may reflect infection-related reactions, and the lack of sustained (i.e., over several cycles) increases of hs-CRP suggested no clinical concern of toxicity. A reduction of cystatin C levels is thought to predict the formation of aneurysms and was therefore measured beginning with Part A cohort 3 onward (18). The use of this plasma assay in conjunction with the radiographic assessment of the large vessels using contrast CT scans represented an additional risk evaluation in patients treated with LY2157299. A sustained reduction in cystatin C levels that may have indicated the development of aneurysms was not apparent for any patient, whether dosed with monotherapy or in combination with lomustine (Fig. 4).

### Electrocardiogram

**Fig. 5:**
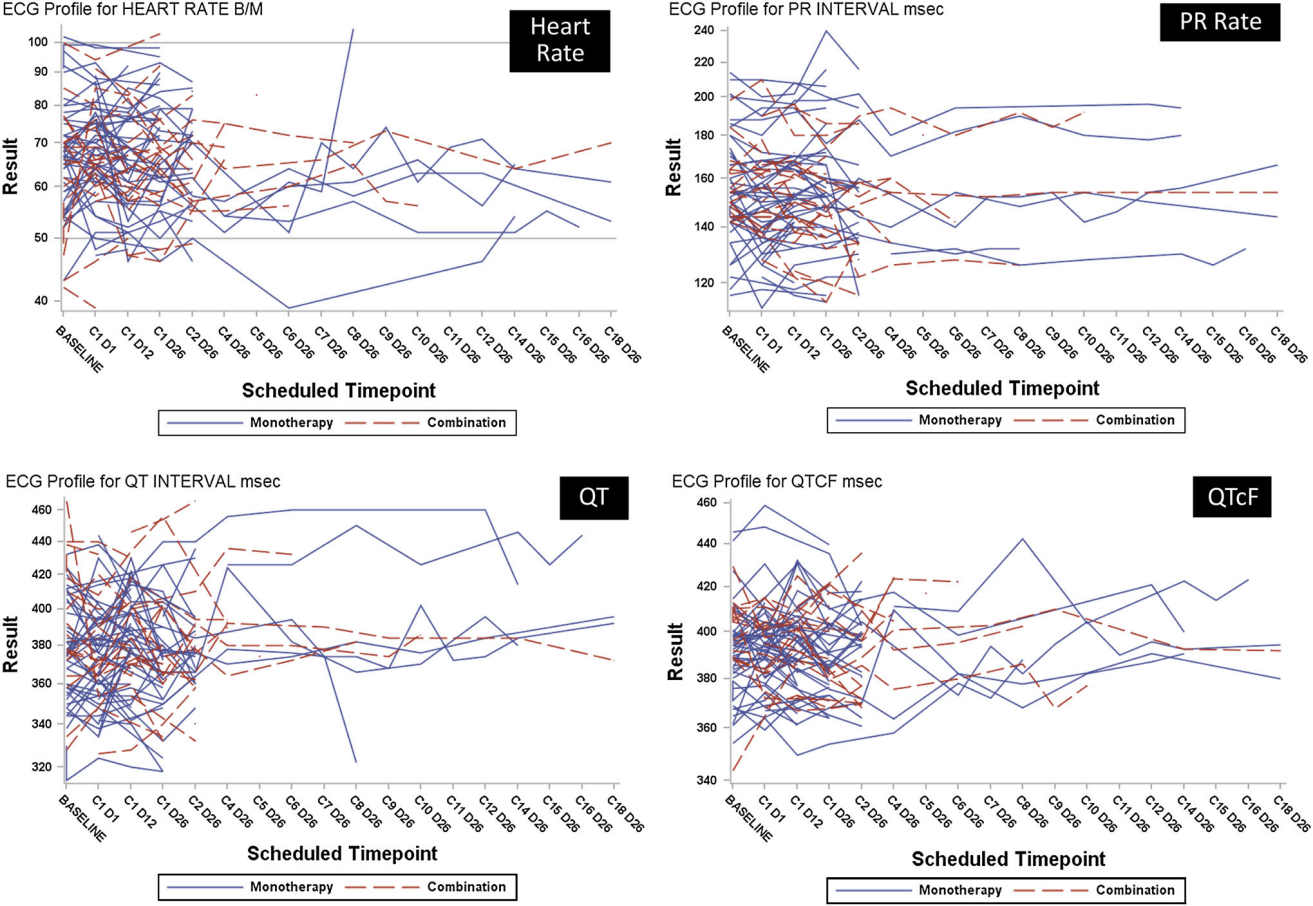
Select electrocardiogram (ECG) parameters. Heart rate as assessed at time of ECG readings. PR rate, QT, and QTcF. *Blue lines* represent patients who received LY2157299 as monotherapy and *red* for patients who were treated with the combination of LY2157299 and lomustine

As part of the comprehensive cardiac safety evaluation of LY2157299, electrophysiological changes were monitored by local and central assessments by cardiologists. Based on the preclinical studies (in vitro and animal in vivo studies), LY2157299 was not associated with QTc prolongation, but this additional monitoring was performed to complement the other cardiac monitoring examinations. We obtained complete ECG readings in 60 patients (60/79; 76 %) and normal ECG readings were observed in 50 % (30/60) at baseline. There was a change to abnormal at least once during treatment in approximately one-third of all patients (Table 4). For patients who continued on treatment, there was no general increase in QTcF or PR rate (Fig. 5). A change of >30 ms in QTcF (Fridericia) was observed in 10/44 patients (22.7 %) during monotherapy treatment (Table 4). There was no patient who had a QTcF prolongation of more than 60 ms or had an excursion over 500 ms. LY2157299 plasma concentration and ECG measurements were not time-matched; however, 16 % of observations were within 10 min, 33 % were within 30 min, 54 % within 1 h and 99 % within 2 h of each other. We used information from a predictive population PK model [14], simulating the individual patient plasma concentration at the exact time of ECG measurement to investigate whether there was a potential for LY2157299 to be associated with QTcF prolongation. Based on this model, we were reassured that the concentrations in humans were not likely to be associated with any QTcF prolongation (data not shown).

**Table 4:** Summary of changes from baseline in overall electrocardiogram assessments and in QTc (Fridericia)

Monotherapy		
*Baseline*		
Missing or normal ECG	Abnormal ECG	
22/42 (52 %)	20/42 (48 %)	
*Change from baseline*		
Normal ECG to abnormal	Abnormal to normal	
11/52 (26 %)	1/42 (2 %)	

Combination		
*Baseline*		
Normal ECG	Abnormal ECG	
8/18 (44 %)	10/18 (56 %)	
*Change from baseline*		
Normal ECG to abnormal	Abnormal to normal	
6/18 (33 %)	0/18 (0 %)	

Increase QTcF interval	Monotherapy *n*/*N* (%)	Combination *n*/*N* (%)
0 and 30 ms	30/44 (68)	16/19 (84)
30 and 60 ms	10/44 (23)	3/19 (16)
>60 ms	0/44 (0)	0/19 (0)

Only includes patients who have both baseline and post-dose electrocardiogram measurements

*ECG* electrocardiogram

### Computer Tomography Scan of the Upper Thorax

Using CT imaging, we did not find any evidence of changes to the ascending aorta or aortic arch.

### Blood Pressure

**Fig. 6:**
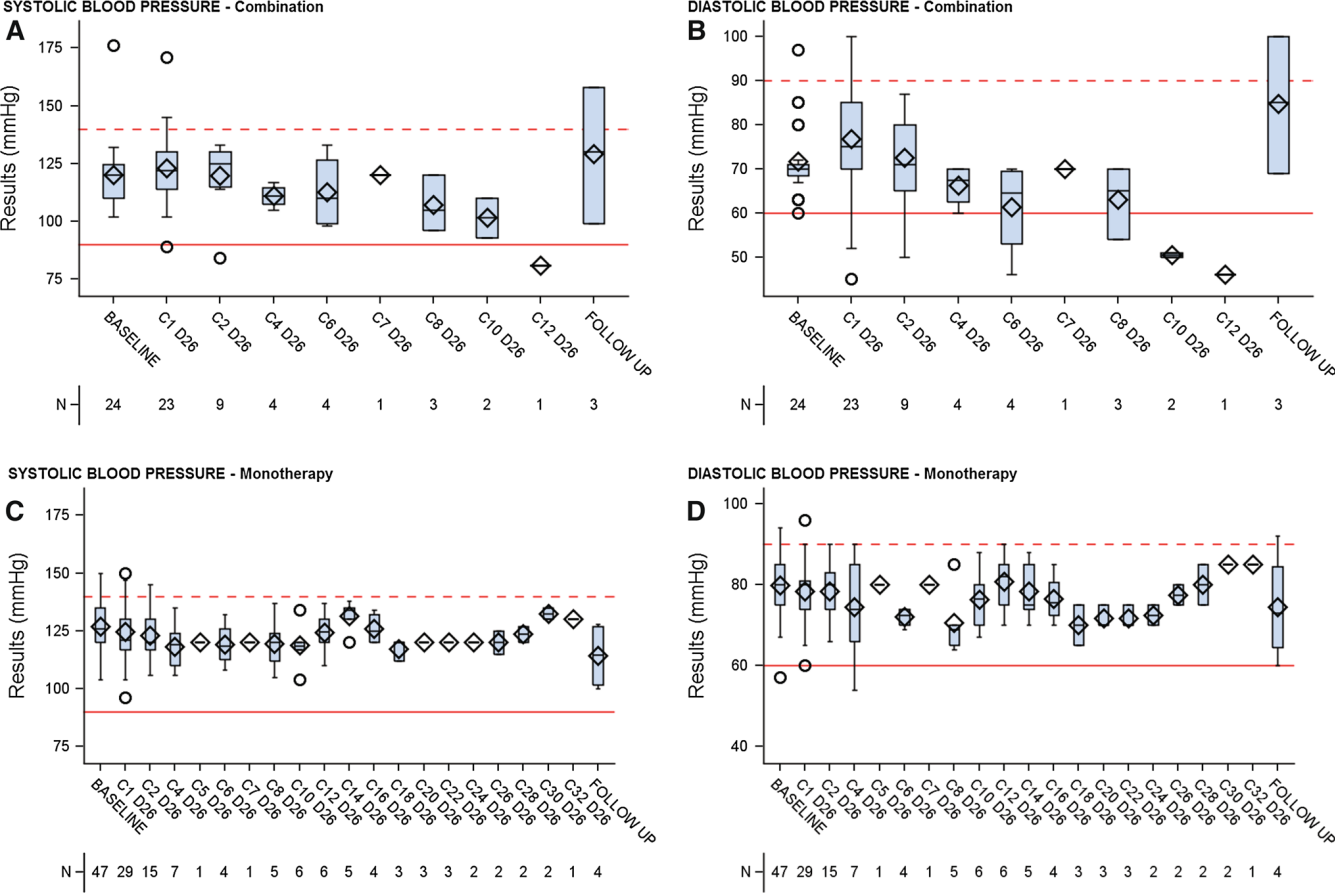
Blood pressure changes over time after the first dose of LY2157299 using cuff-measurements (*horizontal lines* represent normal ranges). Systolic and diastolic blood pressure values over time during the long-term treatment for patients receiving LY2157299 monotherapy [systolic blood pressure (**c**); diastolic blood pressure (**d**)] and combination of LY2157299 and lomustine [systolic blood pressure (**a**); diastolic blood pressure (**b**)]

Blood pressure was evaluated after the first administration during Cycle 1 in Parts A and B of the study. This assessment was designed to determine whether there was an immediate, short-term effect of LY2157299 on blood pressure. Short-term evaluation for Part C was not carried out. During this short-term evaluation (during the first hours post-LY2157299 administration), no changes in systolic and diastolic blood pressure were observed (Fig. 6). LY2157299 had no influence on blood pressure as described for anti-angiogenic compounds.

### Echocardiography/Doppler

**Fig. 7:**
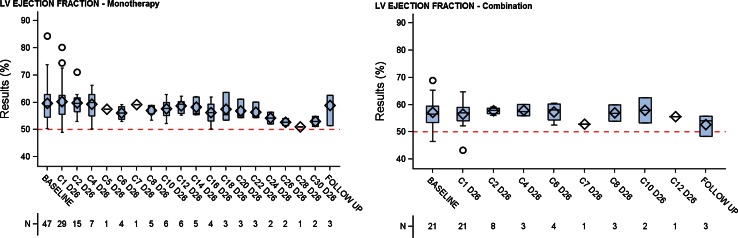
Left ventricular (LV) ejection fraction for monotherapy LY2157299 and combination of LY2157299 and lomustine (*red line* represents the normal value and *open circles* are patients outside of the confidence intervals)

The transthoracic 2D echocardiography/color Spectral Doppler examination assessed overall cardiac structure and function, and potential toxicity risks related to damage to the heart valves, myocardium, or pericardium. The left ventricular function was not changed, including in patients who received treatments longer than six cycles (Fig. 7). In 15 patients, there was a change from baseline that was considered per protocol and central review as potentially pathological, because the valve function suggested a change from a lower risk to a moderate risk. Another patient experienced a change by two severity grades (absent to mild) (Table 5). All these changes were not considered medically significant, and the patient who experienced a change of two severity levels had no other signs of cardiac injury, including plasma BNP or troponin I levels. In this patient, a pulmonary embolism was detected, which may contribute to the changes observed in the echocardiography/Doppler imaging.

**Table 5:** Listing of patients whose aortic, mitral, and tricuspid valve assessment by deterioration of at least 1 category of severity

Patient	Visit	Valvular assessment (aortic = A, mitral = M, tricuspid = T)				Systolic blood pressure (mmHg)
		Absent	Trace	Mild		
R4	Baseline	A, M	T			140
	1		**M**			141
R30	Baseline	A, M	T			104
	1–14		**M**	**T**		104–138
	14		**M**	**T**		138
R5	Baseline	A, M		T		135
	1–2		**M**			110–120
	2		**M**			110
R6	Baseline	A, M, T				130
	1–2		**T**			140–150
	2		**T**			140
R7	Baseline	A, M	T			120
	1–2		**M**			110–140
	2		**M**			110
R12	Baseline	A, T	M			140
	1–2			**M**	***T***	125–130
	2			**M**	***T***	130
R20	Baseline	A, M	T			136
	1–2		**M**			129–134
	2	*M*				129
R21	Baseline	A, M	T			126
	1–31		**M**			105–135
	31	*M*				135
R26	Baseline	A, M, T				134
	1–2		**M**			124–130
	2		**M**			130
R41	Baseline	A, M, T				120
	1–2		**M**, **T**			130
	2	*M*	**T**			130
R42	Baseline	A	M, T			120
	1–8			**M**		117–125
	8			**M**		120
R53	Baseline	A	T	M		176
	1			**T**		158
R54	Baseline	A	M, T			106
	1–10			**T**		84–105
	10		*T*			93
R62	Baseline	A, M, T				110
	1–12		**M**, **T**			81–110
	12		**M**, **T**			81
R65	Baseline	A	T	M		117
	1–4			**T**		120–130
	4			**T**		120

Italics decreased by 1 grade; bold increased by 1 grade; bold italics increased by 2 grades

## Discussion

We report on a prospective and comprehensive cardiac monitoring strategy to assess potential cardiac toxicities for the TGF-β inhibitor LY2157299. Because of the toxicities observed in animals with TGF-β RI inhibitors, we established a cardiac monitoring approach that would detect early signs of cardiac dysfunction. The cardiac toxicity in animals was focused on two major toxicities: (a) valvular changes with localized inflammatory infiltrates and (b) aneurysms observed in dogs and rats treated with LY2157299 continuously for 6 months. In oncology, cardiac toxicity assessments have become increasingly important as preventative strategies are needed to reduce chemotherapy-related cardiac toxicities [20]. Despite this need to assess cardiac safety comprehensively, clinical trials in cancer patients seem to continue to underestimate the risk of cardiac toxicity [21]. This discrepancy may have two main reasons: (1) Toxicities in animal toxicology studies are viewed as not consistently predictive for humans; (2) cardiac toxicities are seen only after several years post-treatment as observed in survivors of pediatric cancer [22]. With treatments containing anthracyclines and bevacizumab, cardiac toxicities are observed in a continuum of time, some occur during the treatment and other patients exhibit the toxicity after treatment [23, 24]. In contrast to such variable or delayed toxicities, LY2157299 or similar TGF-β RI small molecular inhibitors are associated with an immediate cardiac toxicity in animals. Hence, the implementation of comprehensive cardiac toxicity monitoring during the FHD study was imperative. While we expected that the anticipated therapeutic window would not be associated with cardiac toxicity, we needed to collect sufficient data to prove that the chosen therapeutic window was safe. We combined imaging assessments such as CT scans every 6 months for aneurysm evaluation, and echocardiography/Doppler every 2 months for valve formation, with plasma/serum cardiac risk markers.

Echocardiography/color Spectral Doppler was used to monitor possible structural and functional changes of the heart valves [17]. Because TGF-β signaling and serotonin up-regulation in valvular toxicities have been associated, we used echocardiography/color and Spectral Doppler monitoring because it had been useful in identifying the serotonin-induced valve changes [25], in addition to monitoring potential toxicity to myocardial ventricular function. Alternatively, we considered the use of MRI [26], but decided against it because of the patient population and the need to compare results from various study sites. However, echocardiography/Doppler requires that patients are appropriately positioned and cooperative with the examination. In patients with end-of-life conditions, this may sometimes cause compliance issues; hence, the quality of the images can be sub-optimal. Images were generally of good quality, judged both by local review and by the central lab over read. Dual reading is advantageous in a multi-site study, by providing a local review for immediate patient safety, and a central review for consistent quantitation. A disadvantage for the central reviewer is the lack of important clinical information that generally helps in assessing the significance of abnormalities. For example, approximately 70 % of the patients had minimal or small pericardial effusions per central review, deemed medically insignificant by the local cardiologist.

Biomarkers such as BNP and troponin I allowed more frequent monitoring than echocardiography/Doppler. They are used during chemotherapy to detect potential cardiac toxicities [27]. Hence, there is a good understanding on their levels in cancer patients. We observed that none of the patients had an increase of troponin I above 1.0 µg/L. There were some patients who had a slight elevation of about 0.6 µg/L at the start of the trial and also occasionally during the treatment. However, this either did not increase or returned to norm values. This observation is consistent with other tumor types [28] and the slight increases in troponin I are seen in inflammatory conditions [29]. Recent guidelines provide recommendations on the interpretation of troponin elevations in different diseases [30]. The BNP values after age adjustment were generally within the norm values prior to the treatment and in most patients remained unchanged or even decreased. Serial BNP and troponin I levels supported the echocardiography/Doppler findings as previously described in patients with heart failure [31]. The use of hs-CRP was intended to detect inflammatory conditions that may have been induced by blocking the TGF-β secretion. In toxicology studies of the rat with LY2157299, aneurysms were associated with inflammatory cell infiltrates. Hence, the hs-CRP was used as a possible early detection signal [32]. Except for occasional increases in some patients that were associated with infection, the hs-CRP did not increase over time. The regular assessment of cystatin C was implemented as a potential marker for early detection in aneurysms because its reduction over time is associated with abdominal aneurysms [18]. The cystatin C levels were unchanged throughout the treatment with a few reductions in patients treated with LY2157299. In some patients with low pre-treatment levels, there were occasional reductions. However, in such patients or all other patients, the CT scans did not detect aneurysms in the thoracic aorta.

Although LY2157299 treatment was not associated with changes in the HERG-assay or in ECG evaluations in animals, we used ECG as an additional measure for cardiac safety. In general, about half of the patients had an abnormal ECG at baseline that did not change during treatment. This proportion of abnormal baseline ECGs is expected in a sample of adult patients in a clinical trial. In about one-third of the patients, the ECG readings changed from normal to abnormal. The frequencies of patients with a QTc increase of more than 30 ms were about 20 %; no patient had a prolongation of more than 60 ms. After excluding influence factors such as co-medications with known QTc prolongation and considering the comorbidities of patients, there was no indication that LY2157299 induced QTc prolongation.

During treatment with LY2157299, the cardiac health of the glioma patients was unremarkable; LVEF remained unchanged and the blood pressure remained stable. The cardiac medication remained unaltered and patients did not require additional heart medication during the trial. This is also reflected in the serious adverse event/AE reports; no cardiac toxicities were reported. The case of one ischemic event happened after the patient had undergone a re-resection of his tumor and thus was attributed to the surgery. Pulmonary embolism and dyspnea were considered to be associated with the disease as previously reported [33].

As part of the cardiovascular monitoring, all patients had to report the intake of LY2157299 in relationship to food. Because of the stringent administration requirements, patients administered LY2157299 early in the morning (8:00 ± 1 h) and early evening (18:00 ± 1 h). Because of this stringent administration schedule, diurnal fluctuations in cardiac monitoring were assessed and not observed.

An additional benefit of the comprehensive cardiovascular evaluation in this study is to confirm the stability of cardiac function, biomarkers and ECG parameters in sample of very ill cancer patients. Future studies of other cancer therapies can benchmark against these findings, allowing development teams to better determine whether an observed deterioration of cardiac function is due to drug or to the natural history of a late stage cancer.

Based on the lack of any medically significant cardiac events during the administration of LY2157299 either as a short-term or long-term treatment (including in patients treated over 3 years), either the therapeutic window was accurately predicted by the PK/PD model or the preclinical data in rats and dogs were not as predictive. Recently, studies were published that suggest TGF-β is regulated differently in inbred rats, some of which are used in the preclinical assessment of toxicity risk [34]. Furthermore, if shear/stress is a key inducer for TGF-β signaling [35], a block of this pathway may be resulting in more evident toxicity in species where the intravascular pressure is higher than in humans.
